# Nitrogen Starvation Induced Oxidative Stress in an Oil-Producing Green Alga *Chlorella sorokiniana* C3

**DOI:** 10.1371/journal.pone.0069225

**Published:** 2013-07-16

**Authors:** Yun-Ming Zhang, Hui Chen, Chen-Liu He, Qiang Wang

**Affiliations:** 1 Key Laboratory of Algal Biology, Institute of Hydrobiology, Chinese Academy of Sciences, Wuhan, Hubei Province, China; 2 University of Chinese Academy of Sciences, Beijing, China; Laurentian University, Canada

## Abstract

Microalgal lipid is one of the most promising feedstocks for biodiesel production. *Chlorella* appears to be a particularly good option, and nitrogen (N) starvation is an efficient environmental pressure used to increase lipid accumulation in *Chlorella* cells. The effects of N starvation of an oil-producing wild microalga, *Chlorella sorokiniana* C3, on lipid accumulation were investigated using thin layer chromatography (TLC), confocal laser scanning microscopy (CLSM) and flow cytometry (FCM). The results showed that N starvation resulted in lipid accumulation in *C. sorokiniana* C3 cells, oil droplet (OD) formation and significant lipid accumulation in cells were detected after 2 d and 8 d of N starvation, respectively. During OD formation, reduced photosynthetic rate, respiration rate and photochemistry efficiency accompanied by increased damage to PSII were observed, demonstrated by chlorophyll (Chl) fluorescence, 77K fluorescence and oxygen evolution tests. In the mean time the rate of cyclic electron transportation increased correspondingly to produce more ATP for triacylglycerols (TAGs) synthesis. And 0.5 d was found to be the turning point for the early stress response and acclimation of cells to N starvation. Increased level of membrane peroxidation was also observed during OD formation, and superoxide dismutase (SOD), peroxide dismutase (POD) and catalase (CAT) enzyme activity assays suggested impaired reactive oxygen species (ROS) scavenging ability. Significant neutral lipid accumulation was also observed by artificial oxidative stress induced by H_2_O_2_ treatment. These results suggested coupled neutral lipid accumulation and oxidative stress during N starvation in *C. sorokiniana* C3.

## Introduction

Extensive utilization of fossil fuels has led to global climate change, environmental pollution, health problems and an energy crisis, associated with irreversible depletion of traditional sources of fossil fuels [1]. Many countries are thus turning their attention to the development of new, clean, and sustainable energy sources [2]. Biofuel is expected to play a crucial role in the global energy infrastructure in the future, which would bring several benefits such as foreign oil independence, carbon neutral processes, and profits to local farmers [3]. Biodiesel, one of the most commonly used biofuels, has attracted much attention in recent years and is recognized as an ideal renewable energy carrier, and thus also as a possible primary energy source [4]. A variety of biolipids can be used to produce biodiesel, and vegetable oils are a renewable and potentially inexhaustible source of energy with an energy content close to diesel fuel [5]. However, extensive use of vegetable oils may cause significant problems such as starvation in developing countries, and it is important that productive and cultivated land is used for food instead of fuel production. In recent years, renewed interest in producing biodiesel from microalgae has arisen, as microalgae can grow rapidly and convert solar energy into chemical energy via CO_2_ fixation and are thus now considered as one of the most promising sources of oil for making biodiesel [6], [7].

The green microalga *Chlorella* (Chlorophyta), which consists of about 10 species that can grow photoautotrophically, mixotrophically and heterotrophically with high biomass concentration, appears to be a particularly good option for biodiesel production [8]. The oil content in some species of *Chlorella* varies from about 14 to 63% of dry weight, and the fatty acid composition has been reported to range from C-14∶0 to C-20∶0 [9], [10].

Lipid accumulation occurs within the microalgal cells, and varies with growth conditions. N limitation or N starvation is an efficient environmental pressure used to increase lipid accumulation [11]. The general principle is that when there is insufficient N for protein synthesis required for growth, excess carbon from photosynthesis is channeled into storage molecules such as triglyceride or starch [12]. It has been reported that the lipid content in *Chlorella* could be doubled or even tripled under N depletion conditions [13], [14], and a linear relationship between the N source concentration and the lipid content was observed [15]. In addition to the increase in total lipid content in microalgal cells as a result of cultivation in N depleted media, it was found that changing from normal nutrient to N depleted media will gradually change the lipid composition from free fatty acid-rich lipid to mostly triglyceride-containing lipid [16]. Therefore, N limitation or N starvation could increase both lipid and triglyceride content in microalgal cells.

Defined as the third generation biofuel, algal feedstock has become one of the most promising resources for biodiesel production, due to the much higher photosynthetic efficiency to produce biomass, thus resulting in a much higher growth rate and productivity as compared to conventional crops [7], [17]. However, the physiological changes, especially photosynthesis during lipid accumulation and OD formation in oil-producing microalgae under N starvation have not yet been fully elucidated. In this study, an oil-producing microalga isolated from the wild, *Chlorella sorokiniana* C3, was tested to investigate the effects of N starvation on lipid accumulation and photosynthetic parameters. The results showed that N starvation resulted in neutral lipid accumulation accompanied by increased damage to PSII, and an increased level of membrane peroxidation which occurred during OD formation.

## Materials and Methods

### Growth Conditions, N− and Exogenous Oxidative Stress Treatment

The N-sufficient medium (N+) used was full-strength BG11 medium [18]. The N-deficient medium (N−) was BG11 without NaNO_3_. *C. sorokiniana* C3 in the exponential phase was inoculated into a 1 liter Erlenmeyer flask containing 500 ml BG11 medium at 20°C with continuous illumination of 70 µmol m^−2^ s^−1^ and continuously bubbled with filtered air, the initial OD_700_ is 0.05. For N− treatment, cells were harvested by centrifugation at 6,000×g for 3 min at 20°C when they reached the midlogarithmic growth phase (OD_700_ approximately 0.8), and were then washed and resuspended in N− medium to OD_700_ 0.3. For exogenous oxidative stress treatment, cells at midlogarithmic growth phase were harvested by centrifugation at 6,000×g for 3 min at 20°C and were then washed and resuspended to OD_700_ 0.3 with N+ medium including 1.5 mM H_2_O_2_ as the oxidant.

### TLC Analysis of Lipid

10 ml culture at OD_700_ = 1 was harvested at 6,000×g for 3 min, and the cell pellet was washed with fresh medium and centrifuged again. The harvested cell pellet was resuspended in 400 µl of methanol:chloroform mixture (1∶1, v/v). The mixture was shaken for 2 minutes followed by phase separation using 120 µl of 1 M potassium chloride in 0.2 M phosphoric acid. Then the mixture was centrifuged at 12, 000×g at room temperature for 5 min, and the chloroform phase was transferred to a glass tube and dried under nitrogen. The residue was resuspended in a volume of 20 µl chloroform to get the lipid extracts. TLC analysis of lipid extracts from whole cells was performed according to Reiser and Somerville [19] with some modifications. TAGs were separated by developing the plates in hexane-ethyl ether (7.5∶2.5, v/v). Samples were visualized by exposure to iodine vapor for approximately 10 min. 3 µl of each samples extracted at different time points were used for TLC analysis. Glyceryl trioleate (3 µl, 10 mg ml^−1^) was used as a reference substance for TAGs, and the neutral lipid content of *C. sorokiniana* C3 was then determined accordingly by using ImageJ (ver1.41, NIH) [20] and calculated as a percentage of dry cell weight [21].

### CLSM Analysis

Microscopic analysis of the cells was carried out using a confocal scanner (Zeiss LSM 710 NLO). The generation of transmission micrographs for visualization of non-fluorescent protoplast structures was achieved using the manufacturer’s filter settings. A lipophilic fluorescent dye, Bodipy 505/515 (4,4-difluoro-1,3,5,7-tetramethyl-4-bora-3a, 4a-diaza-sindacene; Invitrogen Molecular Probes, Carlsbad, CA, USA), was used to stain the intracellular oil-containing organelles, known as lipid bodies, with a final labeling concentration of 1 µM and 0.1% DMSO (v/v), according to Cooper et al. [22]. Bodipy fluorescence (green) was excited with an argon laser (488 nm) and detected at 505–515 nm. Autofluorescence (red) of algal chloroplasts was detected simultaneously at 650–700 nm.

### FCM Analysis

Samples stained with Bodipy 505/515 were analyzed on a board using a FACSAria flow cytometer (Becton Dickinson, San Jose, CA, USA) equipped with a laser emitting at 488 nm and an optical filter FL1 (530/30 nm). The collected data were analyzed using FlowJo software (Tree Star, San Carlos, CA, USA).

### Pigments Quantification

100% methanol was used to extract the pigments, and the concentrations were determined spectrophotometrically and calculated using the formula developed by Lichtenthaler [23] : Chlorophyll *a* (Chl *a*) (µg ml^−1^) = 16.72 A_665.2_−9.16 A_652.4,_ chlorophyll *b* (Chl *b*) (µg ml^−1^) = 34.09 A_652.4_−15.28 A_665.2_, total chlorophylls (Chl *a*+*b*) (µg ml^−1^) = 1.44 A_665.2_+24.93 A_652.4_, total carotenoids (Car) (µg ml^−1^) = (1000 A_470_ −1.63 Chl *a* –104.96 Chl *b*)/221.

### Photosynthetic Oxygen Evolution and Dark Respiration Rates

Rates of steady state photosynthetic oxygen evolution and respiration were measured using a Clark-type oxygen electrode (Oxylab 2, Hansatech, UK) at 20°C as described by Gao and Xu [24]. Cell suspensions (2 ml) were illuminated at a quantum flux density of 300 µmol m^−2^ s^−1^.

### Chl Fluorescence Analysis

Chl fluorescence was measured using the Dual-PAM-100 Chl fluorescence photosynthesis analyzer (Walz, Germany). Cells were fully dark-adapted for 15 min prior to initial (F_0_) and maximum (F_m_) fluorescence level measurements. The maximum and effective quantum yields of PSII electron transport were calculated as F_v_/F_m_ = (F_m_−F_0_)/F_m_ and F_v_′/F_m_′ = (F_m_′−F_0_′)/F_m_′, respectively, according to Genty et al. [25]; the proportion of closed PSII center, 1−qL, a parameter estimating the fraction of closed PSII center or excitation pressure of PSII was based on a lake mode and was calculated as 1−qL = 1−[(F_m_′−F′)/(F_m_′−F_0_′)×(F_0_′/F′)] [26]; the non-regulated energy dissipation Y(NO) was calculated as Y(NO) = F/F_m_ [26]. Transient increases in Chl fluorescence after turning off actinic light (AL) were monitored according to Shikanai et al. [27].

### 77K Fluorescence

Thylakoid membrane preparation and the 77K fluorescence emission spectra were performed as described previously [28] using a liquid nitrogen device attached to a PTI Fluorometer (QM-4CW, Photon Technology International Inc., South Brunswick, NJ, USA). Both the excitation and emission slit widths were 1 nm, and thylakoid membranes were adjusted to a chlorophyll concentration of 15 µg ml^−1^. Excitation wavelengths of 435 nm and 480 nm were used to excite chlorophyll and carotenoids, respectively.

### Lipid Peroxidation Assessment and ROS Scavenging Enzyme Activity Assays

Malondialdehyde (MDA) level, CAT, POD and SOD activities were measured according to Shi et al. [29]. MDA, CAT and SOD kits were purchased from the Beyotime Institute of Biotechnology, China. The POD kit was purchased from the Nanjing Bioengineering Institute, China.

### Statistical Analyses

Each result shown is the mean of at least three biological replicates. Statistical analysis of the data was performed using the program SPSS-13 and significance was determined at 95% or 99% confidence limits.

## Results

### N starvation Stimulates Lipid Accumulation in *C. sorokiniana* C3

**Figure 1:**
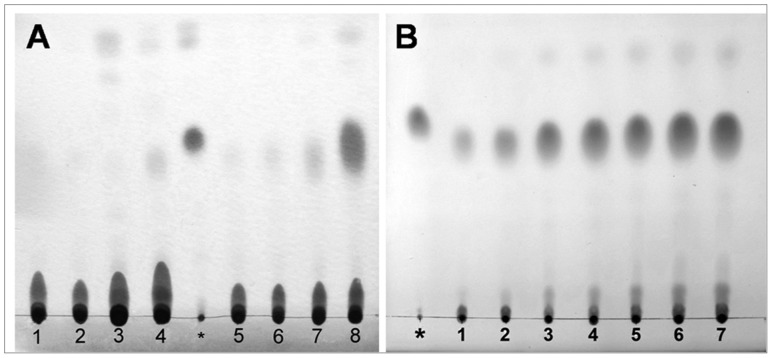
TLC analysis of storage lipid accumulation in *C. sorokiniana* C3 of N+ or N− medium. A, Lanes 1–4, cells in N+ medium at 0 d, 0.5 d, 2 d and 8 d after being resuspended, respectively; asterisk symbol, glyceryl trioleate; lanes 5–8, cells in N− medium at 0 d, 0.5 d, 2 d and 8 d after being resuspended, respectively; B, lanes 1–7, cells in N− medium at 2 d-8 d after being resuspended, respectively; asterisk symbol, glyceryl trioleate.

To determine the regular patterns of lipid accumulation and OD formation in *C. sorokiniana* C3, the intracellular lipid levels at various times of N starvation were examined using TLC. During the first 2 days of treatment, cells resuspended in either N+ (Fig. 1A, lanes 1, 2) or N− medium (Fig. 1A, lanes 5, 6) did not accumulate detectable levels of neutral lipid. When the treatment was prolonged, cells cultured in N− medium started to accumulate neutral lipid after 2 days (Fig. 1A, lane 7), and which rose gradually with the stress time (Fig. 1B). A significant accumulation was observed after 8 days of treatment (Fig. 1A, lane 8 and Fig. 1B, lane 7), and the neutral lipid content got to 18.7% of dry cell weight. In contrast, even after 8 days, only a trace amount of neutral lipid was detected in cells resuspended in N+ medium, and the neutral lipid content was only 2.4% of dry cell weight (Fig. 1A, lane 4). Thus, 0 d, 0–0.5 d, 0.5–2 d and 2–8 d after N starvation were defined as the control stage (Cs), pre-oil droplet formation stage (PDFs), oil droplet formation stage (ODFs), and late-oil droplet formation stage (LDFs), respectively, in further tests.

### Cell Morphology was Affected during OD Formation

**Figure 2:**
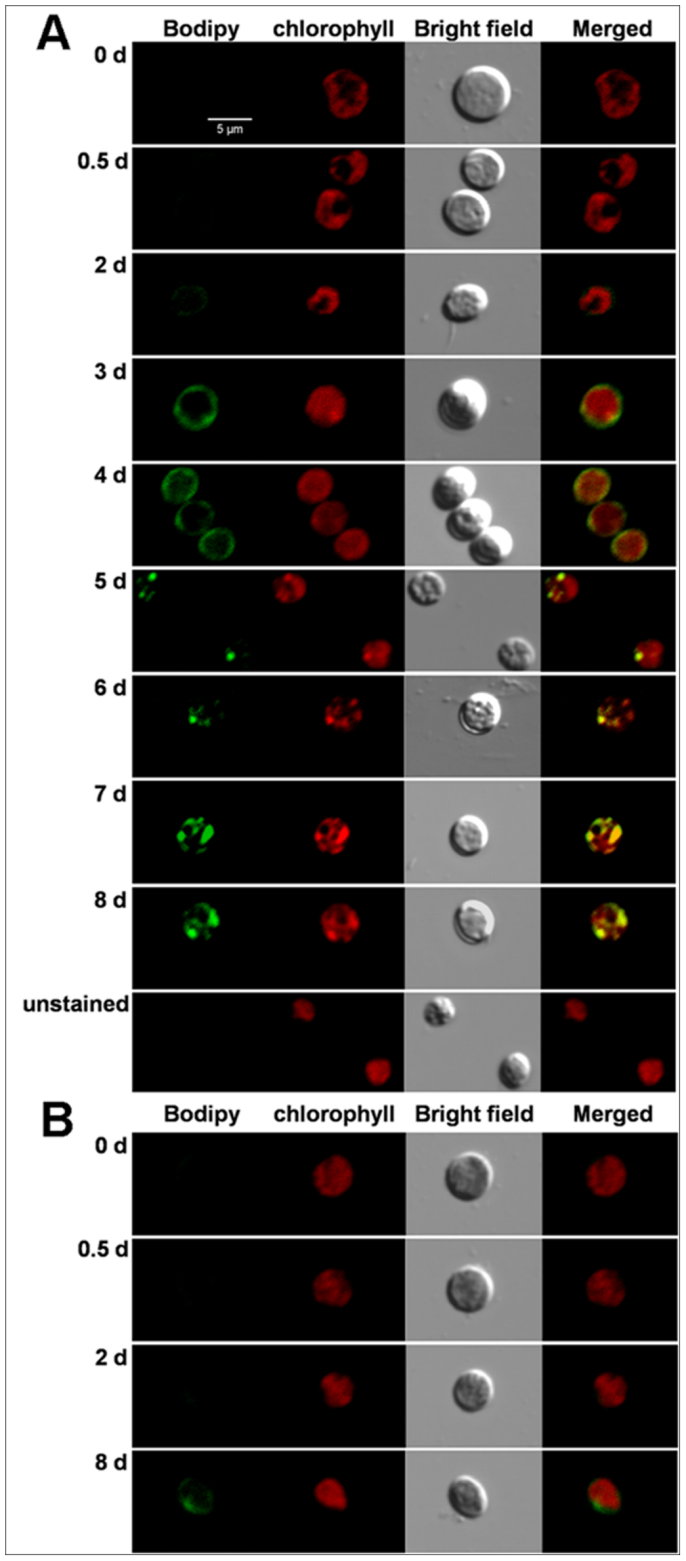
Representative confocal laser scanning micrographs of *C. sorokiniana* C3 labeled *in vivo* with Bodipy 505/515. Bodipy 505/515 (green) was excited with an argon laser (488 nm) and detected at 505–515 nm. Chl autofluorescence (red) was detected simultaneously at 650–700 nm. A, the stained cells resuspended in N− medium for 0 d, 0.5 d, 2 d-8 d, and the unstained cells resuspended in N− medium for 8 d; B, the stained cells resuspended in N+ medium for 0 d, 0.5 d, 2 d and 8 d. The size of the scale bar is shown directly in the image.

To visualize the four key stages of oil-droplet formation, *C. sorokiniana* C3 cells stained with Bodipy 505/515 were observed by CLSM. In accordance with the TLC results (Fig. 1), no Bodipy 505/515 fluorescence (green) was detected at the Cs (Fig. 2A, 0 d) and PDFs (Fig. 2A, 0.5 d), a weak green fluorescence was first detected at the ODFs (Fig. 2A, 2 d), the continuously enhanced green fluorescence signal was detected with time prolonging (Fig. 2A, 3 d-8 d), and a strong green fluorescence signal was observed at the LDFs (Fig. 2A, 8 d). However, only a weak green fluorescence was detected at 8 d in cells in N+ medium, which was much later (Fig. 2B, 8 d). In addition, Chl autofluorescence (red) intensities in cells in both N+ and N− medium were almost identical, whereas Chl autofluorescence at the LDFs showed a significant heterogeneity (Fig. 2). Cell morphology observed by CLSM showed that cell size decreased during N starvation (Fig. 2A). Cells at the Cs showed a variety of cell sizes, suggesting that cells were actively cycling. In contrast, cells under N starvation were relatively concentrated in size, suggested that N starvation inhibited the cell cycle, similar to prochlorophytes [30]. Although cell size was concentrated slightly after 8 days, cells in N+ medium have no significant variation in size (Fig. 2B).

**Figure 3:**
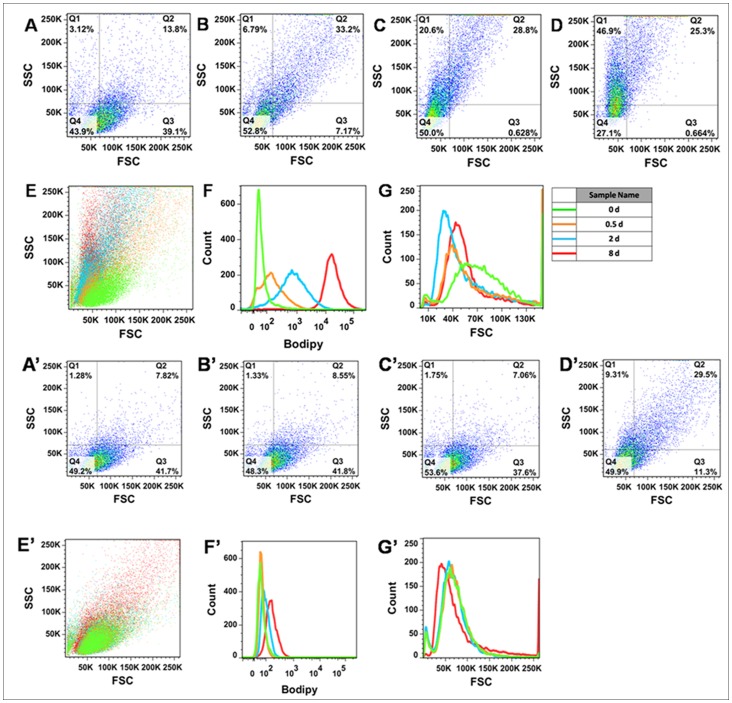
Flow cytometric analysis of *C. sorokiniana* C3 labeled *in vivo* with Bodipy 505/515.

To further characterize the lipid accumulation and cell morphology during N starvation, large numbers of *C. sorokiniana* C3 cells (>10000) at the four stages were analyzed using FCM. Fluorescence intensity of Bodipy 505/515 in cell populations at the PDFs, ODFs and LDFs increased 1.8-fold, 15.0-fold and 262.8-fold, respectively, compared with the Cs, indicating a constant increase in cell neutral lipid content (Fig. 3F). In contrast, no significant difference of fluorescence intensity of Bodipy 505/515 could be observed in cells in N+ medium before 2 days followed a slight increase after 8 days (Fig. 3F′). In FCM, forward scatter (FSC) is normally assumed to be proportional to cell size or cell volume, because the signal intensity increases linearly with the square of the cell diameter to cross-sectional area [31]. At the Cs, FSC was distributed widely, whereas FSC distribution at other stages was more concentrated (Fig. 3A–E, G). The means of FSC at the Cs, PDFs, ODFs, and LDFs were 74.1 K, 72.9 K, 59.8 K, and 62.6 K respectively, which showed that cell volume tended to decrease after N starvation. In contrast, Latasa and Berdalet [32] reported that the cell size of *Heterocapsa* sp. increased under N starvation, whereas there was no clear differences in cell size in the marine diatom, *Ditylum brightwellii,* during N starvation [33]. The side scatter (SSC) of FCM has been shown to be affected by cell morphology, and especially by intracellular structures that are determined by chemical composition (e.g. starch content) [34]. A higher SSC intensity is usually obtained from cells with a higher level of cytoplasmic granularity [35]. An increase in SSC was detected, the means of which at the Cs, PDFs, ODFs, and LDFs were 50.7 K, 86.5 K, 99.9 K, and 122 K, respectively (Fig. 3A–E), indicating increased cell morphological complexity caused by damage due to N starvation stress. However, the changes of both FSC and SSC were minor in cells in N+ medium, and only small differences could be detected even after 8 days (Fig. 3A′–E′, G′). These results suggested that N starvation had an adverse effect on *C. sorokiniana* C3 cell survival and growth, and the alga accumulated and stored lipids in cells for recovery and re-growth under the appropriate conditions.

### Photosynthetic Pigments Composition during OD Formation

Although lipid accumulation can be accelerated by N starvation, as a stressor, N starvation can also lead to depression of photosynthesis. To evaluate the variation in *C. sorokiniana* C3 photosynthesis during OD formation, pigment contents were determined spectrophotometrically. As shown in Table 1, Chl *a*, Chl *b*, Chl *a*+*b* and Car contents in cells in N+ medium all increased with time, and the variation in Chl *a*/Chl *b* and Car/Chl *a*+*b* were minor. In contrast, Table 1 indicates that there were significant changes in pigment composition in cells in N− medium during OD formation. Chl *a*, Chl *a*+*b* and Car contents in cells appeared to be similar, and all had a transient increase at the PDFs and then decreased, whereas Chl *b* content was inversely correlated to N starvation time. Chl *a*/Chl *b* increased up to the ODFs, which indicated reduced chloroplast photosynthetic phosphorylation activity [36] and acclimation of photosynthesis when first subjected to the stress condition.

**Table 1:** Variation in pigment contents in *C. sorokiniana* C3 cells during OD formation.

		Chl *a* (µg ml^−1^)	Chl *b* (µg ml^−1^)	Chl *a*+*b* (µg ml^−1^)	Car (µg ml^−1^)	Chl *a*/*b*	Car/Chl *a*+*b*
Cs		4.881±0.183	1.721±0.251	6.602±0.708	1.242±0.147	2.994±0.753	0.188±0.040
PDFs	−N	5.402±0.539	1.496±0.247	6.900±0.786	1.564±0.170	3.641±0.336	0.227±0.010
	+N	8.324±0.773	2.894±0.029	11.219±0.755	2.119±0.297	2.876±0.465	0.189±0.012
ODFs	−N	4.608±0.323	1.119±0.224	5.726±0.547	1.379±0.125	4.119±0.772	0.241±0.023
	+N	11.077±0.628	3.930±0.187	15.007±0.781	3.071±0.245	2.819±0.266	0.205±0.009
LDFs	−N	2.671±0.115	0.798±0.080	3.470±0.194	0.743±0.038	3.348±0.385	0.214±0.036
	+N	15.810±0.634	6.504±0.450	22.313±1.057	4.823±0.161	2.431±0.274	0.216±0.007

Values are mean ± SD (t test, n = 3–5, P<0.05).

### Steady State Photosynthetic Rate and Dark Respiration Rate during OD Formation

**Figure 4:**
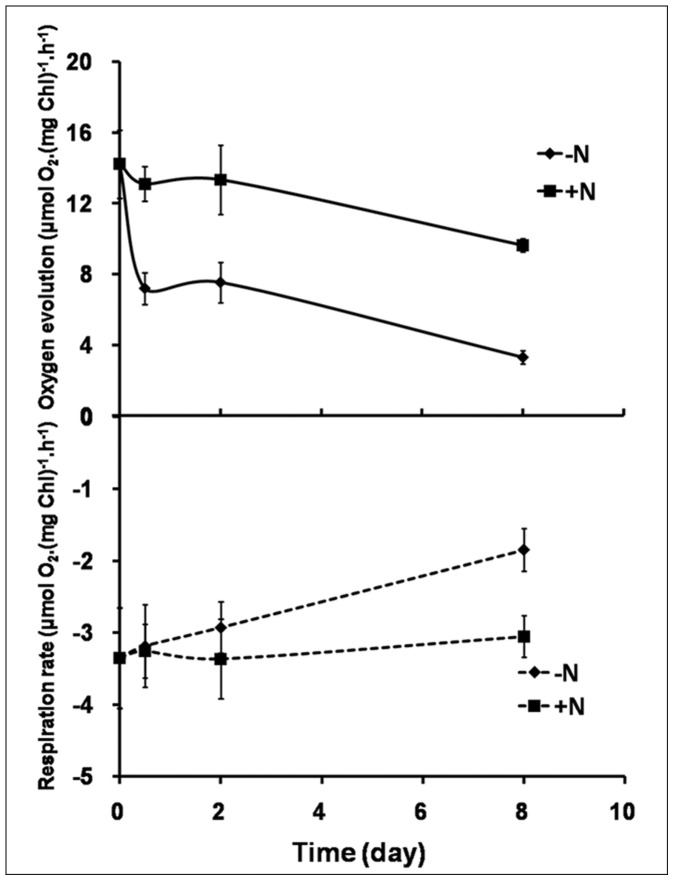
Variations in steady-state oxygen evolution and dark respiration in *C. sorokiniana* C3 during OD formation. Solid line, oxygen evolution. Dotted line, respiration rate. Black square symbols, cells resuspended in N+ medium. Solid up arrow symbols, cells resuspended in N− medium. Values are means ± SD (n = 3). When error bars cannot be seen, error is less than the size of the symbol.

To further investigate the variation in photosynthesis of *C sorokiniana* C3 during OD formation, the steady state photosynthetic rate and dark respiration rate of cells were examined. Compared with cells cultured in N+ medium, both steady state photosynthetic rate and dark respiration rate of cells under N starvation were reduced significantly (Fig. 4). As shown in Figure 4, photosynthetic oxygen evolution rate dropped sharply to 50.7% at the PDFs, and remained at the same level until the ODFs, then decreased to 23.6% at the LDFs, indicating that permanent damage to photosynthetic apparatus had occurred. In addition, an approximately linear decrease in dark respiration rate during OD formation was observed, and statistical analysis showed that the dark respiration rate had a significant inversely correlation with OD formation (correlation test, r = −0.978<0, P<0.05).

### Chl Fluorescence Change Under N Starvation

**Figure 5:**
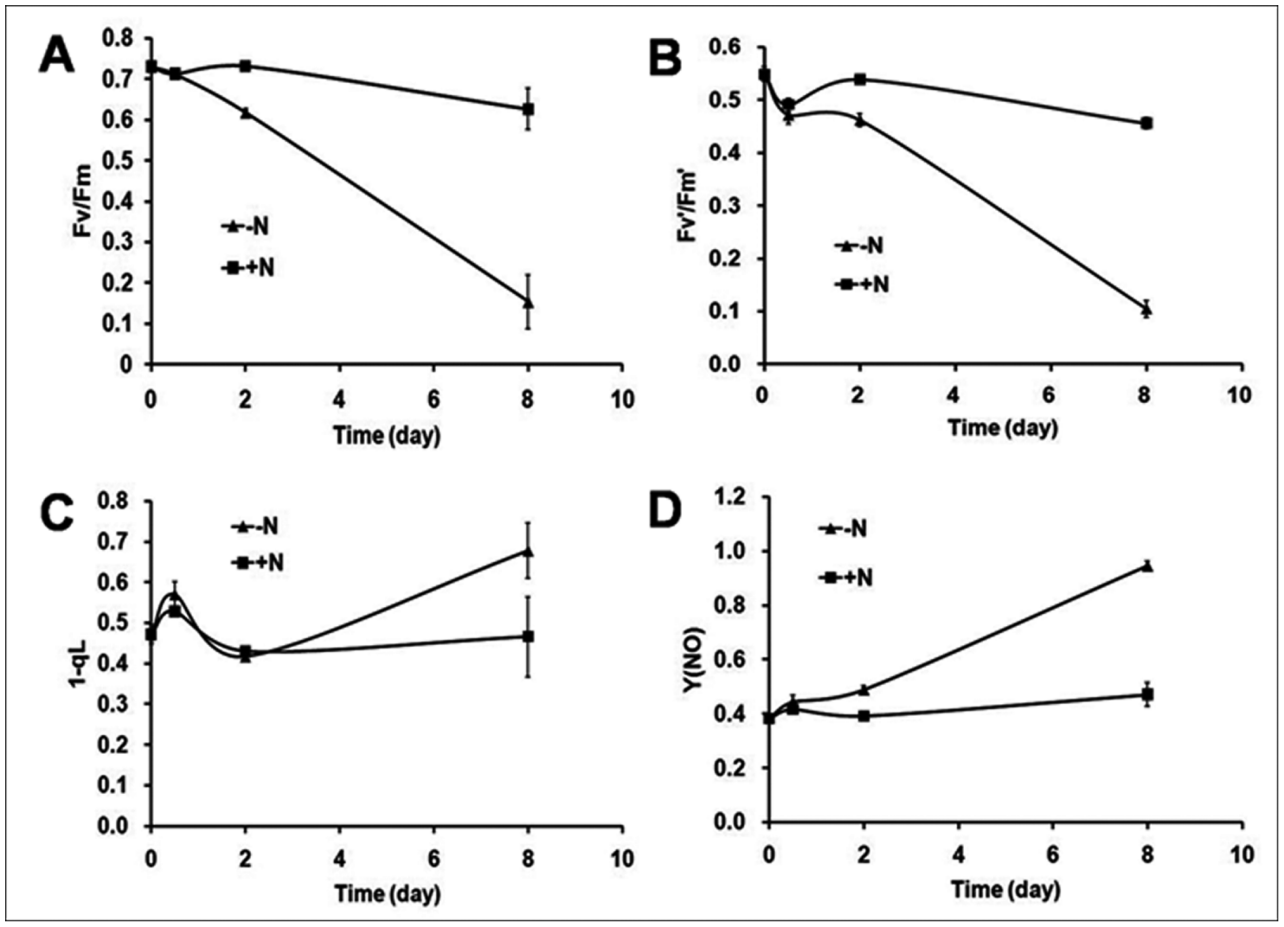
Chl fluorescence parameters of *C. sorokiniana* C3 during OD formation. Chl fluorescence of cells was monitored with a Dual-PAM-100 fluorimeter. Black square symbols, cells resuspended in N+ medium. Solid up arrow symbols, cells resuspended in N− medium. A, Fv/Fm, B, Fv′/Fm′, C, 1-qL, D, Y(NO). Measurements were performed in darkness at room temperature and saturation pulses were applied every 30 s. Data are shown as the means ± SD (n = 3).

To gain more insight into the photosynthetic activities of *C sorokiniana* C3 during OD formation, various parameters of PSII activity were determined using variable fluorescence measurements. In microalgae, nutrient stress is generally detected by a decrease in F_v_/F_m_ [37], and the F_v_/F_m_ value in cultures without stress has been found to be relatively constant, but decreases in cultures under nutrient stress. Figure 5A shows that the maximum photochemical efficiency of PSII (F_v_/F_m_) declined linearly during OD formation (negative correlation by correlation test, r = −0.992<0, P<0.01). Although the effective quantum yields of PSII (F_v_′/F_m_′) declined with stagnation at the PDFs and ODFs (Fig. 5B), statistical analysis also showed a significant negative correlation between F_v_′/F_m_′ and OD formation (correlation test, r = −0.986<0, P<0.05). A decrease in both F_v_/F_m_ and F_v_′/F_m_′ indicated increasing damage to PSII. 1-qL, a parameter which estimates the fraction of closed PSII center or excitation pressure of PSII [26], increased 20.76% at the PDFs, but decreased by 26.84% at the PDFs and subsequently at the ODFs, and then increased again at the LDFs (Fig. 5C), suggesting an immediate response, acclimation, and permanent damage to PSII at the PDFs, ODFs and LDFs, respectively, which corresponded with the pigments analysis and oxygen evolution test results, but appeared to be more sensitive. In contrast, an increase in damaging nonphotochemical quenching Y(NO), an indicator of the extent of damage to PSII [26], was observed, which was positively correlated to OD formation (correlation test, r = 0.992>0, P<0.01 ) (Fig. 5D). Different from the cells cultured in N− medium, F_v_/F_m_, F_v_′/F_m_′, 1-qL and Y(NO) in cells cultured in N+ medium were relatively stable among all stages (Fig. 5).

**Figure 6:**
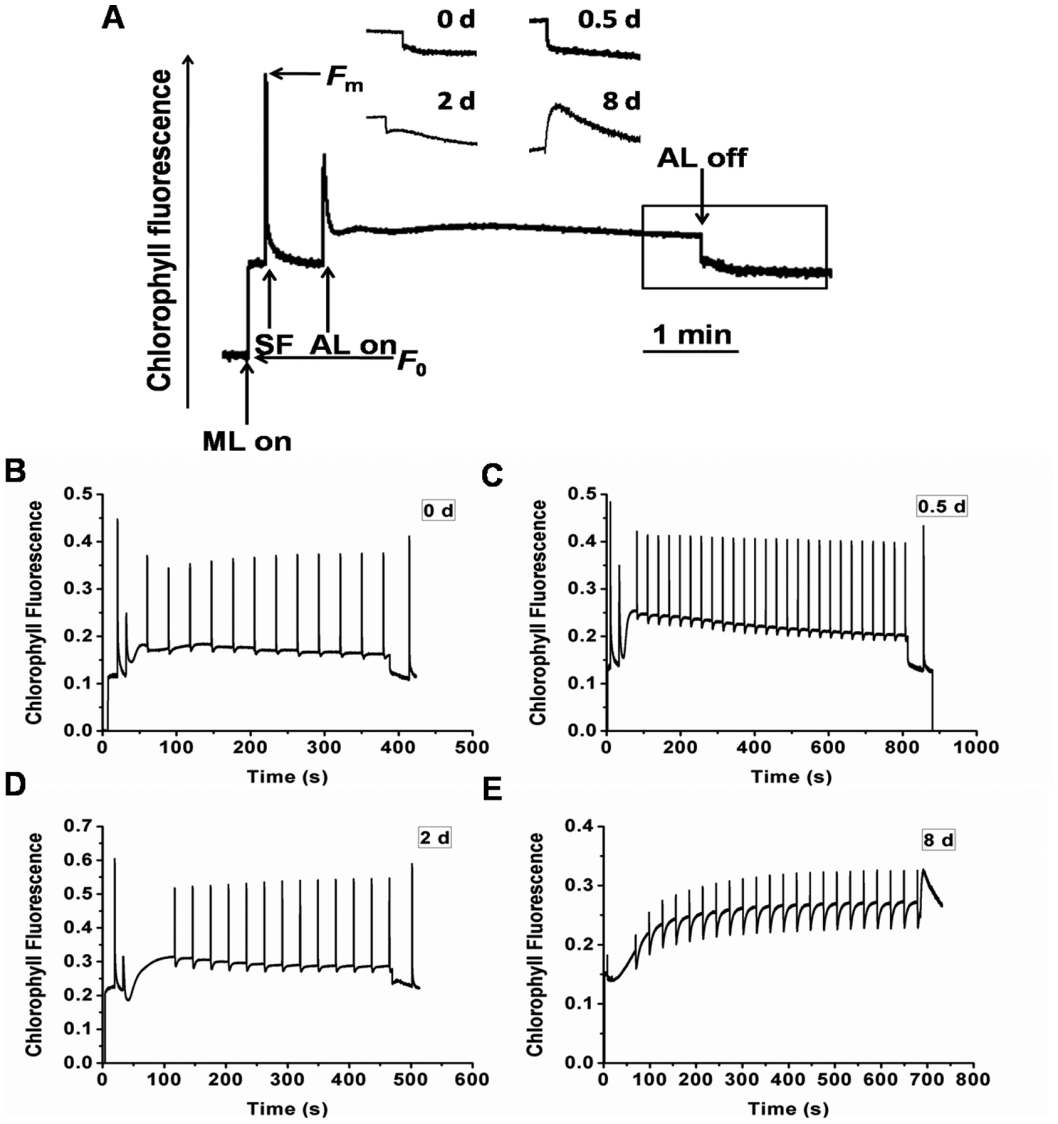
Characterization of Chl fluorescence in *C. sorokiniana* C3 during OD formation. A, Transient post-illumination increase in Chl fluorescence. ML, modulating light beam. SF, saturating flash of white light. The curve shows a typical trace of Chl fluorescence in the cells on 0 d. Cells were exposed to actinic light (AL) (70 µE) for 5 min. AL was turned off and the subsequent change in Chl fluorescence level was monitored. Chl fluorescence image was captured at the times indicated by the scale bar. After switching the AL off, transient increases in Chl fluorescence were recorded under low measure light. Insets are magnified traces from the boxed area. B–E, Chl fluorescence induction traces of *C. sorokiniana* C3 on 0 d, 0.5 d, 2 d and 8 d respectively. Measurements were performed in darkness at room temperature and saturation pulses were applied every 30 s. Data are shown as the means ± SD (n = 3).

The post-illumination transient increase in Chl fluorescence represents the NAD(P)H dehydrogenase (NDH)-dependent reduction in the plastoquinone pool in darkness and was considered to be a measurement of the cyclic electron flow [27]. A typical induction curve of Chl fluorescence in *C. sorokiniana* C3 is shown in Figure 6A, and the representative induction curves at different stages are shown in Figure 6B–E. As shown in the inserts of Figure 6A, the transient post-illumination increase in Chl fluorescence was undetectable at the Cs and PDFs, while a slight and a remarkable increase were detected at the ODFs and LDFs, respectively. All these results indicated increasing cyclic electron flow around PSI with prolonged N starvation, which supplies ATP to TAG synthesis when the rate of both photophosphorylation and respiratory oxidative phosphorylation dropped (Fig. 4).

### 77K Fluorescence Spectroscopy Under N Starvation

**Figure 7:**
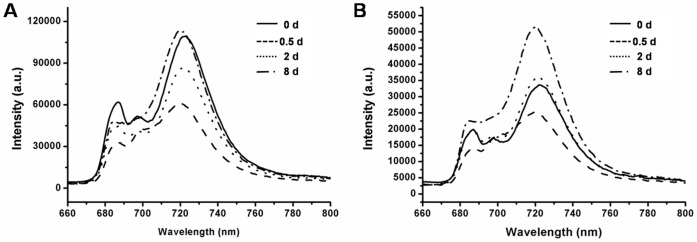
77K fluorescence of thylakoid membranes of *C. sorokiniana* C3 during OD formation. Fluorescence emission spectra of thylakoid membranes of *C. sorokiniana* C3 under N starvation for 0 d, 0.5 d, 2 d and 8 d. Excitation wavelengths were: 435 nm (A), 480 nm (B). After dark adaptation, cells were frozen and the spectra were recorded at 77K. All samples correspond to 15 µg ml^−1^ Chl.

The impact of N starvation on excitation energy distribution and the stoichiometry of photosystems were also diagnostically investigated by 77K fluorescence spectroscopy. Thylakoid membranes were isolated from cells cultured in N− medium at the four stages, and 77K fluorescence emission spectra were recorded in liquid nitrogen with excitation wavelengths at 435 nm (Fig. 7A) and 480 nm (Fig. 7B), to excite Chl and Car, respectively, and both showed very similar patterns.

The typical emission spectra of the Cs thylakoids showed a major peak at 723 nm (*F*723), which corresponded to PSI, and two smaller peaks at 687 nm (*F*687) and 697 nm (*F*697), which originated mainly from PSII. When normalized at 687 nm, both the amplitude and bandwidth of the PSI fluorescence (*F*723) increased simultaneously with treatment time (Fig. 7A and 7B), and a 3 nm blue shift (from 723 nm to 720 nm) was observed. No peak shifts were detected in PSII bands (*F*687 and *F*697), except for the PDFs (*F*687 blue shifted to 685 nm, *F*697 red shifted to 702 nm), furthermore, there was a dramatic increase in the amplitude of *F*697 at the PDFs. These exceptions at the PDFs corresponded with the pigments (Table 1) and Chl fluorescence (Fig. 5) results, suggesting that the PDFs could be the turning point for the early stress response and acclimation to N starvation. The *F*PSI/*F*PSII ratio generally correlates well with the relative content of PSI and PSII [38], interestingly, it was found that when excited at either 435 nm or 480 nm, the *F*PSI/*F*PSII ratios were significantly positively correlated to OD formation (correlation test, 435 nm, r = 0.971>0, P<0.05; 480 nm, r = 0.985>0, P<0.05, respectively). The increased *F*PSI during N starvation correspondence with the increased cyclic electron flow around PSI measured by chlorophyll fluorescence transient (Fig. 6).

### Lipid Peroxidation and Activities of the ROS Scavenging Enzymes

**Figure 8:**
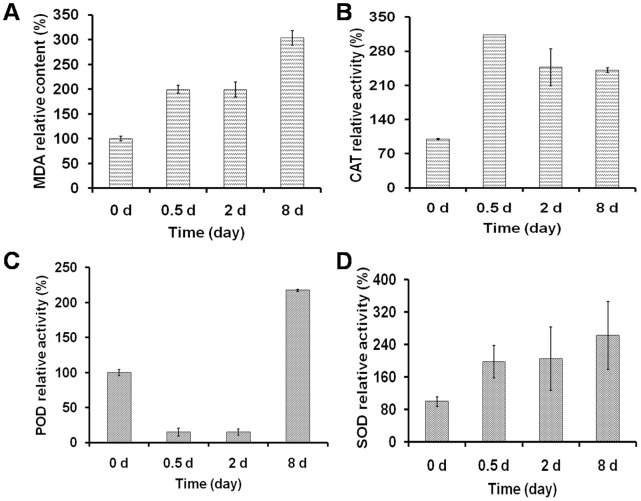
Lipid peroxidation level and antioxidant enzymes activities of *C. sorokiniana* C3 during OD formation. A–D represent the MDA content, CAT, POD and SOD activities, respectively. Data are shown as the means ± SD (n = 3).

In oxygenic photosynthetic organisms, most stress conditions could ultimately be described as oxidative stress [39]. Lipid peroxidation, the most commonly accepted indicator of oxidative stress, was estimated by measuring the contents of MDA in cells during OD formation. As shown in Figure 8A, although stagnation between 0.5 d and 2 d was detected, N starvation induced a significant increase in the MDA level in cells, indicating serious membrane system damage during OD formation caused by N starvation-induced oxidative stress.

During oxidative stress, ROS, including ^•^O_2_^−^, ^1^O_2_ and H_2_O_2_, with corresponding levels will be generated [40], and the scavenging enzymes including SOD, CAT, and POD will be induced or activated in cells [41]. Figure 8 shows that the relative activities of SOD increased during OD formation and tripled at the LDFs, i.e. 8 days after N starvation. The relative activities of CAT increased to 312.94% at the PDFs and dropped slightly to 241.16% at the LDFs. In contrast, the relative activities of POD declined first to 14.91% and 15.02% at the PDFs and ODFs, respectively, and then dramatically increased to 217.44% at the LDFs. Therefore, SOD, CAT and POD played important roles in removing ROS in *C. sorokiniana* C3 under N starvation at different stages.

### Exogenous Oxidative Stress Improves Lipid Accumulation in *C. sorokiniana* C3

**Figure 9:**
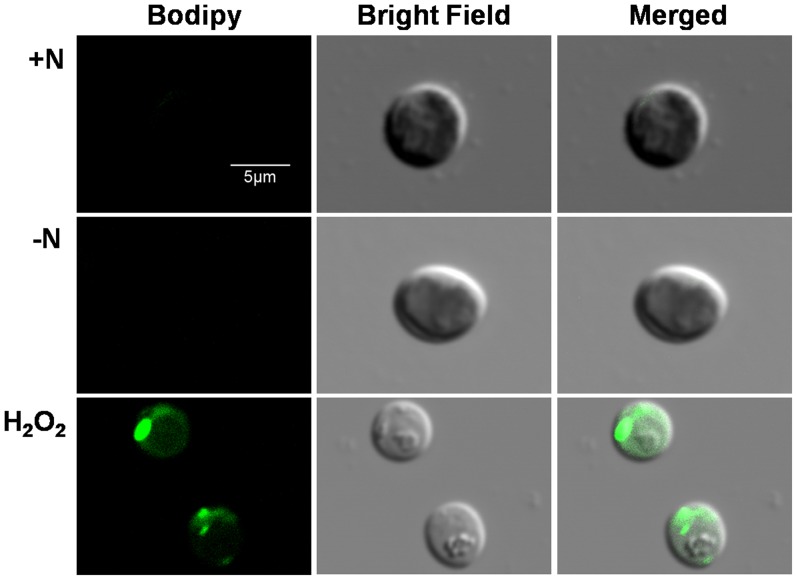
Representative confocal laser scanning micrographs of *C. sorokiniana* C3 labeled *in vivo* with Bodipy 505/515 under N+, N− or exogenous oxidative stress condition. Bodipy 505/515 (green) was excited with an argon laser (488 nm) and detected at 505–515 nm. All cells were detected at 1 d after being resuspended in N+, N− or N+ plus H_2_O_2_ medium, respectively. The size of the scale bar is shown directly in the image.

To further confirmed the possible connection between oxidative stress and neutral lipid accumulation, H_2_O_2_ was used as the exogenous oxidant and neutral lipid accumulation in cells was detected by using CLSM with Bodipy 505/515. As shown in Figure 9, after 1 d treatment, neutral lipid was undetectable in cells under both N+ and N− conditions, while a significant accumulation was observed under exogenous oxidative stress induced by H_2_O_2_, indicated that exogenous oxidative stress could be a more effective factor for neutral lipid induction in *C. sorokiniana* C3.

## Discussion

Whether conditions are favorable or adverse, the intricate metabolic pathways of a cell are heavily influenced by its environment. In the case of microalgae, specialized cultivation can stimulate changes in metabolism [42]. It has been reported that N starvation enhances TAGs accumulation in many microalgal strains such as *Neochloris oleoabundans*, *Chlorella vulgaris* and *Chlorella protothecoides* [43]–[45]. The TLC profiles showed that the green alga *C. sorokiniana* C3 accumulated significant amounts of TAGs after N starvation (Fig. 1). In particular, it was found that in our algal cells, ODs were first detected after 2 days of N starvation (Fig. 1A, lane 7), and significant accumulation was observed after 8 days of treatment (Fig. 1A, Lane 8), which corresponded with our further tests by CLSM (Fig. 2) and FCM (Fig. 3). Thus, 0 d, 0–0.5 d, 0.5–2 d and 2–8 d after N starvation could be defined as the Cs, PDFs, ODFs, and LDFs, respectively, accordingly to the four key stages of lipid accumulation in *C. sorokiniana* C3.

In *C. sorokiniana* strains, various attempts to improve lipid production have been reported during the past few years. By using UV mutagenesis, four *C. sorokiniana* mutants contained elevated total lipid and TAGs content were selected [46]. In particular, it was found that culture condition was important for lipid accumulation and the studied on maximize lipid production by changing culture condition got more attention. For example, a study found that C/N ratios had a significant effect on cell lipid content in heterotrophic *C. sorokiniana*, and low C/N ratios favored high cell lipid content [21]. Wan et al. [47] revealed that a *C. sorokiniana* strain under heterotrophic condition could accumulate 56% (w/w) dry weight lipid content and much higher than photoautotrophic culture. Ngangkham et al. [48] also revealed that *C. sorokiniana* under mixotrophic conditions recorded more lipid productivity. In the present study, 18.7% neutral lipid content was detected in cells under N limitation, which was much lower than heterotrophic or mixotrophic culture cells mentioned above. However, as more cost in microalgae culture under heterotrophic or mixotrophic conditions, photoautotrophic culture for microalgae grow and lipid accumulation is economically more feasible. Thus, the study for improving neutral lipid accumulation in microalgae cells under photoautotrophic condition is critical. In addition, in our previous study the cell viability of *C. sorokiniana* C3 has not reduce significantly under N starvation as compared to N+ culture (data not published), so it was no significant influence on the overall lipid yield as well, which gives this strain an obvious applicable value as a test organism in studying neutral lipid accumulation mechanism under N starvation for improving biofuel production.

Higher ratios of Chl *a*/Chl *b* have been considered to decrease light collection in relation to the rate of PSII photochemistry [36], and Car could have an antioxidative role in protecting unsaturated lipids in oil bodies from peroxidation, and could participate in the screening and trapping of excessive light otherwise absorbed by the chloroplast [49]. In the present study, increased Chl *a*/Chl *b* and Car/Chl *a*+*b* ratios were found in *C. sorokiniana* C3 during N starvation, indicating that the light-harvesting complexes in *C. sorokiniana* C3 cells decreased and led to a decrease in the PSII photochemistry rate, which was further indicated by Chl fluorescence (Fig. 5) and 77K fluorescence (Fig. 7).

During OD formation under N starvation, the efficiency of PSII in *C. sorokiniana* C3 decreased initially, as the consequences of the reduced photosynthetic pigments, the increased proportion of closed PSII center, the reduced light collection capability and the intensified photo damage, all of which induced serious PSII damage, decreased the rate of photosynthesis leading to inhibition of oxygen evolution and respiration rate, and resulted in less ATP synthesis. In the mean time the accumulation of TAGs requires more ATP, thus the rate of cyclic electron flow around PSI increased significantly to compensate (Fig. 6). The up-regulation of PSI fluorescence peak resulted in a higher *F*_723_/*F*_687_ ratio (Fig. 7), further proved that N starvation affected the stoichiometry of the photosystems, and thus the excited energy distribution between the two photosystems. The damage of N starvation to photosynthesis in *C. sorokiniana* has also been reported in a study by Vona et al. [50], in which a marked loss of photosynthetic activity and the lower value of respiratory activity in cells after a 24 h of N starvation were exhibited.

Interestingly, the significant changes at the PDFs could be detected in many photosynthetic parameters. For example, Chl *a*, Chl *a*+*b* and Car contents all showed a transient increase at the PDFs and then decreased (Table 1), and 1-qL increased at the PDFs, but subsequently decreased at the ODFs (Fig. 5C), all suggested that the PDFs could be the turning point for the early stress response and acclimation to N starvation. At the initial stage of N starvation, *C. sorokiniana* C3 tried to acclimate to the stress by regulating photosynthesis after the immediate response, but failed with aggravated stress when the N starvation extended, and thus permanent damage occurred at the later stages. Therefore, the PDFs, ODFs and LDFs during OD formation corresponded to the immediate response, acclimation of *C. sorokiniana* C3 to N starvation stress, and permanent damage to cells, respectively.

The lipid peroxidation, a commonly accepted indicator of oxidative stress, was assayed by measuring the level of MDA. In the present study, it was found that MDA increased significantly in *C. sorokiniana* C3 cells with N starvation during OD formation, which indicated that oxidative stress was generated due to N starvation (Fig. 8). To mitigate and repair the damage caused by ROS, antioxidants including free radical scavenging nonenzymes of low molecular mass (e.g., β-carotene) and enzymes such as SOD, POD, and CAT usually accumulate in cells [51]. In the present study, SOD converted ^•^O_2_^−^ into H_2_O_2_ and O_2_ at all four stages, and CAT scavenged H_2_O_2_ effectively and kept the oxidation damage at a relatively lower level (Fig. 8A) at earlier stages (PDFs and ODFs), but failed at LDFs indicated by a 3 fold increase of the MDA level (Fig. 8A), although the activity of POD increased significantly to compromise. According to the changes of neutral lipid (Fig. 1, Fig. 2A and Fig. 3F) and MDA level (Fig. 8A) during N starvation, a linear relationship could be found between neutral lipid accumulation and the MDA level. These results suggested a possible connection between the N starvation induced oxidative stress and neutral lipid accumulation, which was further confirmed by the result that artificial oxidative stress (H_2_O_2_) could significantly induce neutral lipid accumulation (Fig. 9). Moreover, the H_2_O_2_ induced exogenous oxidative stress seemed like a more effective factor for neutral lipid induction in *C. sorokiniana* C3, 1 d induction by H_2_O_2_ (Fig. 9) resulted even more neutral lipid accumulated in cells than 5 to 6 d under N starvation (Fig. 2A). Considering the time and oil content, exogenous oxidative stress supposed to be a more noteworthy method in the attempts to improve neutral lipid accumulation in microalgae.

In summary, N starvation resulted in neutral lipid accumulation in *C. sorokiniana* C3 cells, and OD formation and significant neutral lipid accumulation in cells occurred on 2 d and 8 d after N starvation, respectively. During OD formation, reduced photosynthetic rate, oxygen evolution, respiration rate and photochemistry efficiency accompanied by increased damage to PSII were observed. In the mean time the cyclic electronic flow increased to produce more ATP for TAGs synthesis, with increased proportion and energy distribution to PSI. The PDFs was considered to be the turning point for the early stress response and acclimation to N starvation, and the PDFs, ODFs and LDFs corresponded to the immediate response, acclimation of cells to stress and the permanent damage to cells during OD formation, respectively. Aroused oxidative stress indicated by increased level of membrane peroxidation was also observed during OD formation, together with the results that exogenous oxidant stress with H_2_O_2_ induced significantly of the neutral lipid accumulation, which suggested a coupling between neutral lipid accumulation and oxidative stress during N starvation. As compared with N starvation, the more effective of neutral lipid induction in cells by exogenous oxidative stress may provide new insights into cost effectively microalgae lipid production.

## References

[1] Amaro HM, Guedes AC, Malcata FX (2011) Advances and perspectives in using microalgae to produce biodiesel. Applied Energy 88: 3402–3410.

[2] Farrell AE, Plevin RJ, Turner BT, Jones AD, O’Hare M, et al (2006) Ethanol can contribute to energy and environmental goals. Science 311: 506–508. 16439656 10.1126/science.1121416

[3] Heredia-Arroyo T, Wei W, Ruan R, Hu B (2011) Mixotrophic cultivation of *Chlorella vulgaris* and its potential application for the oil accumulation from non-sugar materials. Biomass & Bioenergy 35: 2245–2253.

[4] Chisti Y (2007) Biodiesel from microalgae. Biotechnology Advances 25: 294–306. 17350212 10.1016/j.biotechadv.2007.02.001

[5] Demirbas A (2011) Biodiesel from oilgae, biofixation of carbon dioxide by microalgae: A solution to pollution problems. Applied Energy 88: 3541–3547.

[6] Ahmad AL, Yasin NHM, Derek CJC, Lim JK (2011) Microalgae as a sustainable energy source for biodiesel production: A review. Renewable & Sustainable Energy Reviews 15: 584–593.

[7] Mata TM, Martins AA, Caetano NS (2010) Microalgae for biodiesel production and other applications: A review. Renewable & Sustainable Energy Reviews 14: 217–232.

[8] Petkov G, Garcia G (2007) Which are fatty acids of the green alga *Chlorella*? Biochemical Systematics and Ecology 35: 281–285.

[9] O’Grady J, Morgan JA (2011) Heterotrophic growth and lipid production of *Chlorella protothecoides* on glycerol. Bioprocess and Biosystems Engineering 34: 121–125. 20976474 10.1007/s00449-010-0474-y

[10] Gouveia L, Oliveira AC (2009) Microalgae as a raw material for biofuels production. Journal of Industrial Microbiology & Biotechnology 36: 269–274. 18982369 10.1007/s10295-008-0495-6

[11] Rodolfi L, Zittelli GC, Bassi N, Padovani G, Biondi N, et al (2009) Microalgae for oil: strain selection, induction of lipid synthesis and outdoor mass cultivation in a low-cost photobioreactor. Biotechnology and Bioengineering 102: 100–112. 18683258 10.1002/bit.22033

[12] Scott SA, Davey MP, Dennis JS, Horst I, Howe CJ, et al (2010) Biodiesel from algae: challenges and prospects. Current Opinion in Biotechnology 21: 277–286. 20399634 10.1016/j.copbio.2010.03.005

[13] Converti A, Casazza AA, Ortiz EY, Perego P, Del Borghi M (2009) Effect of temperature and nitrogen concentration on the growth and lipid content of *Nannochloropsis oculata* and *Chlorella vulgaris* for biodiesel production. Chemical Engineering and Processing: Process Intensification 48: 1146–1151.

[14] Widjaja A, Chien CC, Ju YH (2009) Study of increasing lipid production from fresh water microalgae *Chlorella vulgaris* . Journal of the Taiwan Institute of Chemical Engineers 40: 13–20.

[15] Hsieh CH, Wu WT (2009) Cultivation of microalgae for oil production with a cultivation strategy of urea limitation. Bioresource Technology 100: 3921–3926. 19362823 10.1016/j.biortech.2009.03.019

[16] Takagi M, Watanabe K, Yamaberi K, Yoshida T (2000) Limited feeding of potassium nitrate for intracellular lipid and triglyceride accumulation of *Nannochloris* sp UTEX LB1999. Applied Microbiology and Biotechnology 54: 112–117. 10952013 10.1007/s002530000333

[17] Chen CY, Yeh KL, Aisyah R, Lee DJ, Chang JS (2011) Cultivation, photobioreactor design and harvesting of microalgae for biodiesel production: A critical review. Bioresource Technology 102: 71–81. 20674344 10.1016/j.biortech.2010.06.159

[18] Stanier RY, Kunisawa R, Mandel M, Cohen-Bazire G (1971) Purification and properties of unicellular blue-green algae (order *Chroococcales*). Bacteriological Reviews 35: 171–205. 4998365 10.1128/br.35.2.171-205.1971 PMC378380

[19] Reiser S, Somerville C (1997) Isolation of mutants of *Acinetobacter calcoaceticus* deficient in wax ester synthesis and complementation of one mutation with a gene encoding a fatty acyl coenzyme A reductase. Journal of Bacteriology 179: 2969–2975. 9139916 10.1128/jb.179.9.2969-2975.1997 PMC179062

[20] Tsihlis ND, Murar J, Kapadia MR, Ahanchi SS, Oustwani CS, et al (2010) Isopropylamine NONOate (IPA/NO) moderates neointimal hyperplasia following vascular injury. Journal of Vascular Surgery 51: 1248–1259. 20223627 10.1016/j.jvs.2009.12.028 PMC2860688

[21] Chen F, Johns MR (1991) Effect of C/N Ratio and Aeration on the Fatty-Acid Composition of Heterotrophic *Chlorella Sorokiniana* . Journal of Applied Phycology 3: 203–209.

[22] Cooper MS, Hardin WR, Petersen TW, Cattolico RA (2010) Visualizing “green oil” in live algal cells. Journal of Bioscience and Bioengineering 109: 198–201. 20129108 10.1016/j.jbiosc.2009.08.004

[23] Lichtenthaler HK (1987) Chlorophylls and carotenoids: Pigments of photosynthetic biomembranes. In: Lester Packer RD, editor. Methods in Enzymology: Academic Press. 350–382.

[24] Gao H, Xu X (2009) Depletion of Vipp1 in *Synechocystis* sp. PCC 6803 affects photosynthetic activity before the loss of thylakoid membranes. FEMS Microbiology Letters 292: 63–70. 19222583 10.1111/j.1574-6968.2008.01470.x

[25] Genty B, Briantais J-M, Baker NR (1989) The relationship between the quantum yield of photosynthetic electron transport and quenching of chlorophyll fluorescence. Biochimica et Biophysica Acta (BBA) - General Subjects 990: 87–92.

[26] Kramer D, Johnson G, Kiirats O, Edwards G (2004) New fluorescence parameters for the determination of Q_A_ redox state and excitation energy fluxes. Photosynthesis Research 79: 209–218. 16228395 10.1023/B:PRES.0000015391.99477.0d

[27] Shikanai T, Endo T, Hashimoto T, Yamada Y, Asada K, et al (1998) Directed disruption of the tobacco *ndhB* gene impairs cyclic electron flow around Photosystem I. Proceedings of the National Academy of Sciences. 95: 9705–9709. 10.1073/pnas.95.16.9705 PMC21403 9689145

[28] Wang Q, Jantaro S, Lu B, Majeed W, Bailey M, et al (2008) The high light-inducible polypeptides stabilize trimeric Photosystem I complex under high light conditions in *Synechocystis* PCC 6803. Plant Physiology 147: 1239–1250. 18502976 10.1104/pp.108.121087 PMC2442545

[29] Shi S, Tang D, Liu Y (2009) Effects of an algicidal bacterium *Pseudomonas mendocina* on the growth and antioxidant system of *Aphanizomenon flos-aquae* . Current Microbiology 59: 107–112. 19365689 10.1007/s00284-009-9404-0

[30] Vaulot D, Partensky F (1992) Cell cycle distributions of prochlorophytes in the north western Mediterranean Sea. Deep Sea Research Part A Oceanographic Research Papers 39: 727–742.

[31] Toepel J, Wilhelm C, Meister A, Becker A, Martinez-Ballesta MdC (2004) Cytometry of freshwater phytoplankton. In: Zbigniew Darzynkiewicz MR, Hans T, editors. Methods in Cell Biology: Academic Press. 375–407. 10.1016/s0091-679x(04)75015-3 15603434

[32] Latasa M, Berdalet E (1994) Effect of nitrogen or phosphorus starvation on pigment composition of cultured *Heterocapsa* sp. Journal of Plankton Research 16: 83–94.

[33] Brussaard CPD, Noordeloos AAM, Riegman R (1997) Autolysis kinetics of the marine diatom *Ditylum Brightwellii* (Bacillariophyceae) under nitrogen and phosphorus limitation and starvation. Journal of Phycology 33: 980–987.

[34] Hyka P, Lickova S, Přibyl P, Melzoch K, Kovar K (2012) Flow cytometry for the development of biotechnological processes with microalgae. Biotechnology Advances, in press. 10.1016/j.biotechadv.2012.04.007 22561949

[35] Davey HM, Kell DB (1996) Flow cytometry and cell sorting of heterogeneous microbial populations: the importance of single-cell analyses. Microbiological Reviews 60: 641–696. 8987359 10.1128/mr.60.4.641-696.1996 PMC239459

[36] Demmig-Adams B, Adams WI (1996) Chlorophyll and carotenoid composition in leaves of *Euonymus kiautschovicus* acclimated to different degrees of light stress in the field. Functional Plant Biology 23: 649–659.

[37] White S, Anandraj A, Bux F (2011) PAM fluorometry as a tool to assess microalgal nutrient stress and monitor cellular neutral lipids. Bioresource Technology 102: 1675–1682. 20965719 10.1016/j.biortech.2010.09.097

[38] Murakami A (1997) Quantitative analysis of 77K fluorescence emission spectra in *Synechocystis* sp. PCC 6714 and *Chlamydomonas reinhardtii* with variable PS I/PS II stoichiometries. Photosynthesis Research 53: 141–148.

[39] Elstner EF (1991) Mechanisms of oxygen activation in different compartments of plant cells. In: Pelland EJ, Steffen KL, editors. Active Oxygen/Oxidative Stress in Plant Metabolism. Rockville, MD: American Society of Plant Physiologists. 13–25.

[40] Apel K, Hirt H (2004) Reactive oxygen species: metabolism, oxidative stress, and signal transduction. Annual Review of Plant Biology 55: 373–399. 10.1146/annurev.arplant.55.031903.141701 15377225

[41] Ali MB, Yu KW, Hahn EJ, Paek KY (2005) Differential responses of anti-oxidants enzymes, lipoxygenase activity, ascorbate content and the production of saponins in tissue cultured root of mountain *Panax ginseng* C.A. Mayer and *Panax quinquefolium* L. in bioreactor subjected to methyl jasmonate stress. Plant Science 169: 83–92.

[42] Rosenberg JN, Oyler GA, Wilkinson L, Betenbaugh MJ (2008) A green light for engineered algae: redirecting metabolism to fuel a biotechnology revolution. Current Opinion in Biotechnology 19: 430–436. 18725295 10.1016/j.copbio.2008.07.008

[43] Illman A, Scragg A, Shales S (2000) Increase in *Chlorella* strains calorific values when grown in low nitrogen medium. Enzyme and Microbial Technology 27: 631–635. 11024528 10.1016/s0141-0229(00)00266-0

[44] Li Y, Horsman M, Wang B, Wu N, Lan C (2008) Effects of nitrogen sources on cell growth and lipid accumulation of green alga *Neochloris oleoabundans* . Applied Microbiology & Biotechnology 81: 629–636. 18795284 10.1007/s00253-008-1681-1

[45] Li Y, Fei X, Deng X (2012) Novel molecular insights into nitrogen starvation-induced triacylglycerols accumulation revealed by differential gene expression analysis in green algae *Micractinium pusillum* . Biomass & Bioenergy 42: 199–211.

[46] Vigeolas H, Duby F, Kaymak E, Niessen G, Motte P, et al (2012) Isolation and partial characterization of mutants with elevated lipid content in *Chlorella sorokiniana* and *Scenedesmus obliquus* . Journal of Biotechnology 162: 3–12. 22480533 10.1016/j.jbiotec.2012.03.017

[47] Wan MX, Wang RM, Xia JL, Rosenberg JN, Nie ZY, et al (2012) Physiological evaluation of a new *Chlorella sorokiniana* isolate for its biomass production and lipid accumulation in photoautotrophic and heterotrophic cultures. Biotechnology and Bioengineering 109: 1958–1964. 22354808 10.1002/bit.24477

[48] Ngangkham M, Ratha SK, Prasanna R, Saxena AK, Dhar DW, et al. (2012) Biochemical modulation of growth, lipid quality and productivity in mixotrophic cultures of *Chlorella sorokiniana*. SpringerPlus: 33. 10.1186/2193-1801-1-33 PMC3725904 23961362

[49] Edge R, McGarvey DJ, Truscott TG (1997) The carotenoids as anti-oxidants – a review. Journal of Photochemistry and Photobiology B: Biology 41: 189–200. 10.1016/s1011-1344(97)00092-4 9447718

[50] Vona V, Rigano VD, Esposito S, Carillo P, Carfagna S, et al (1999) Growth, photosynthesis, and respiration of *Chlorella sorokiniana* after N-starvation. Interactions between light, CO2 and NH4+ supply. Physiologia Plantarum 105: 288–293.

[51] Chen H, Jiang JG (2011) Toxic effects of chemical pesticides (trichlorfon and dimehypo) on *Dunaliella salina* . Chemosphere 84: 664–670. 21621243 10.1016/j.chemosphere.2011.03.032

